# The Quality, Readability, and Accuracy of the Information on Google About Cannabis and Driving: Quantitative Content Analysis

**DOI:** 10.2196/43001

**Published:** 2023-05-02

**Authors:** Maria Josey, Dina Gaid, Lisa D Bishop, Michael Blackwood, Maisam Najafizada, Jennifer R Donnan

**Affiliations:** 1 School of Pharmacy, Memorial University of Newfoundland St. John's, NL Canada

**Keywords:** cannabis, driving, quality, readability, accuracy, public education, internet, Google search, analysis, accessibility, information, evaluation, tool, data, misinterpretation

## Abstract

**Background:**

The public perception of driving under the influence of cannabis (DUIC) is not consistent with current evidence. The internet is an influential source of information available for people to find information about cannabis.

**Objective:**

The purpose of this study was to assess the quality, readability, and accuracy of the information about DUIC found on the internet using the Google Canada search engine.

**Methods:**

A quantitative content analysis of the top Google search web pages was conducted to analyze the information available to the public about DUIC. Google searches were performed using keywords, and the first 20 pages were selected. Web pages or web-based resources were eligible if they had text on cannabis and driving in English. We assessed (1) the quality of information using the Quality Evaluation Scoring Tool (QUEST) and the presence of the Health on the Net (HON) code; (2) the readability of information using the Gunning Fox Index (GFI), Flesch Reading Ease Scale (FRES), Flesch-Kincaid Grade Level (FKGL), and Simple Measure of Gobbledygook (SMOG) scores; and (3) the accuracy of information pertaining to the effects of cannabis consumption, prevalence of DUIC, DUIC effects on driving ability, risk of collision, and detection by law enforcement using an adapted version of the 5Cs website evaluation tool.

**Results:**

A total of 82 web pages were included in the data analysis. The average QUEST score was 17.4 (SD 5.6) out of 28. The average readability scores were 9.7 (SD 2.3) for FKGL, 11.4 (SD 2.9) for GFI, 12.2 (SD 1.9) for SMOG index, and 49.9 (SD 12.3) for FRES. The readability scores demonstrated that 8 (9.8%) to 16 (19.5%) web pages were considered readable by the public. The accuracy results showed that of the web pages that presented information on each key topic, 96% (22/23) of them were accurate about the effects of cannabis consumption; 97% (30/31) were accurate about the prevalence of DUIC; 92% (49/53) were accurate about the DUIC effects on driving ability; 80% (41/51) were accurate about the risk of collision; and 71% (35/49) were accurate about detection by law enforcement.

**Conclusions:**

Health organizations should consider health literacy of the public when creating content to help prevent misinterpretation and perpetuate prevailing misperceptions surrounding DUIC. Delivering high quality, readable, and accurate information in a way that is comprehensible to the public is needed to support informed decision-making.

## Introduction

In October 2018, the use of nonmedical cannabis became legal in Canada [1]. By the end of 2020, approximately 20% of Canadians, aged 15 years and older, reported using cannabis over the previous 3 months [2]. Certain cannabis use behaviors can increase the risk of experiencing harmful effects [3], such as daily use of cannabis, using cannabis products with high tetrahydrocannabinol content, or driving under the influence of cannabis (DUIC) [4]. In Canada, approximately 1-2 out of every 5 cannabis consumers engage in some form of risky behaviors [3], with 4%-12% of all injuries and deaths from motor vehicle accidents being attributed to DUIC [5]. Additionally, 40% of participants in a Canada-wide survey reported riding with a cannabis-impaired driver within the past year [6].

There are mixed perceptions among the general public regarding the true risks associated with cannabis use [7-9]. In particular, the mixed beliefs regarding the risks associated with DUIC are concerning given the potential impact on both the consumer and innocent members of the public. Recent literature reported that perception of risks associated with DUIC is low, with one study reporting that 28% of participants believed there was no increased risk of accidents [6]. Another study reported that of those who participated in DUIC, 43% believed it was not a risky behavior [10]. This highlights the need to ensure cannabis consumers have access to evidence-based information to support informed decision-making [7,11,12].

Although information about cannabis can be retrieved from numerous sources, one study reported that 78% of participants relied on knowledge gained from their own personal experiences, while 39% obtained information from the internet [13]. Cannabis-related Google searches increased by 75% between 2004 and 2016 [14,15]; however, the trustworthiness of information retrieved on the internet is questionable. There have been studies that explored the quality of cannabis labels from products sold on the web [16], the accuracy of cannabis claims on common websites [17], and the quality of cannabis-related information in magazines and newspapers [18,19]. In general, these studies reported that the quality of cannabis-related information were very poor. Among studies that specifically looked at cannabis health claims on the internet, one found only 5% of claims on the health benefits of cannabis aligned with evidence [20]. Other studies reported that web-based information about cannabis use for pain was biased as sources often neglected to discuss potential risks [21] or were just unreliable [22]. This points to variable quality of cannabis-related information available on the internet [20,23-26].

Web-based search trends related to health-related topics provide insight on the public perception or cannabis use, which also reflect the availability of public health resources [27]. Taking into consideration that searches related to cannabis increased by 75% on Google from 2004 to 2016 [14,15], high quality, easily accessible, evidence-based information is needed for individuals to make informed decisions about cannabis use behaviors [28], which is especially important given the prevalent misconceptions about DUIC. However, the quality, readability, and accuracy of information found through the Google search engine on DUIC are still unknown [6]. The purpose of this study was to assess the quality, readability, and accuracy of information about DUIC found on the internet through the Google search engine.

## Methods

### Study Design

A quantitative content analysis about DUIC was performed on public web pages using the Google Canada search engine.

### Eligibility Criteria

To be included, the web page had to (1) have information related to cannabis and driving, (2) be available in English, (3) be accessible with no fee, (4) have text to analyze, and (5) be available at the time of analysis. Web pages were excluded if (1) the page became no longer available during analysis and (2) the web page only contained images.

### Data Collection

Web pages were identified through the Google search engine. Google was chosen because it is the dominant search engine in Canada, holding 91.98% of market shares [29], and one study showed that 89.8% of people preferred using Google [30]. A private search through incognito mode was used to avoid the search history from biasing results. Six separate Google searches were performed using the terms outlined in Textbox 1, and the first 20 URLs were collected from each search. Neutral search terms were used to ensure the collected web pages were not biased in one direction. The first 20 URLs were collected, as most people consider no more than the first 20 web pages when performing an internet search [15,31]. For our study, one researcher (SS or MJ) extracted web page addresses with Google Chrome (version 99.0.4844.51) [32]. The search was first completed in October 2021 using the first 4 search terms and then repeated fully in April 2022 after 2 new search terms were added.

**Search terms used to collect web pages for analysis.**

**Google search terms**

- Cannabis AND driving
- Marijuana AND driving
- Pot AND driving
- Weed AND driving
- Driving high
- Driving stoned

### Data Analysis

Web pages were organized into categories based on categories used in similar studies that assessed the quality of health-related information on the internet [33]. These categories included digital media, commercial web pages, government organizations, health organizations, nonprofit foundations, peer-reviewed materials, and “other.”

### Outcome Measures

#### Quality of Information

The quality of the information was measured by 2 tools: the Health on the Net (HON) code and the Quality Evaluation Scoring Tool (QUEST).

##### HON Code

HON is a nonprofit foundation aimed to assess and evaluate the quality of web-based health information [34]. The HON certification is designed so that people of the general public can identify trustworthy sources of information [34] and has been used in previous research to evaluate health-related websites as a beneficial tool that shows the intent of a website to publish high-quality information [35-38].The HON code seeks to promote trustworthy health information for the benefit of internet users [39]. HON code is a voluntary certification used on health websites, indicating that their 8 principles were fulfilled. Those principles relate to the authority, complementarity, confidentiality, attribution, justifiability, transparency, financial disclosure, and advertisement policy of the website content [40]. This certification aims to certify websites that are reliable and of high quality, so it is an easy measure for the general public to quickly determine if the web page is a trustworthy source of health information.

##### The QUEST Tool

The QUEST tool serves as a standard for assessing the quality of web-based health information that does not rely on users’ subjective judgment [41]. The QUEST tool was chosen as it has been validated and assessed for reliability and provides a numeric score allowing for quantitative analysis [42]. The QUEST tool was validated for both treatment and preventative measures of web-based health care information [41] and has since been used in studies to evaluate web-based health care information on various topics, including papillomavirus and oropharyngeal cancer, COVID-19, and using electronic cigarettes [33,43,44]. Additionally, this tool is used for a broad range of health topics as opposed to more focused health topics (eg, treatment) [41]. The QUEST tool assesses 7 aspects of the website information and provides a weighted score out of 28 (Table 1) [41]. Three independent researchers collaborated to assess the quality of the web page, while each page was assessed by at least two researchers (SS, MJ, MB), and any discrepancies were discussed. For our study, if an organization took ownership over the text (rather than a specific author), we gave a score of 1, meaning “all other indications of authorship” on the QUEST tool scoring. Additionally, any language that promoted the sale of cannabis (eg, cannabis brand) or directed the reader to a specific location for purchase was given a score of 1 accordingly under the “Conflicts of Interest” section of the QUEST scoring tool. For example, any mention of a specific cannabis dispensary, even if indirectly mentioned through a picture identifying a dispensary, was considered an endorsement, and therefore, had the potential to be biased.

**Table 1:** Description of the Quality Evaluation Scoring Tool (QUEST) criteria to evaluate the quality of web-based health information [41]. Scores in the individual sections are weighted and summed to generate a total score of up to 28. This tool is reproduced and distributed under the terms of the Creative Commons Attribution 4.0 International License [45].

Characteristics						Score
**Authorship**						Score x 1
	0: No indication of authorship or username					
	1: All other indications of authorship					
	2: Author’s name and qualification clearly stated					
**Attribution**						Score x 3
	0: No sources					
	1: Mention of expert source, research, research findings (although with insufficient information to identify the specific studies), links to various sites, advocacy body, or other					
	2: Reference to at least one identifiable scientific study, regardless of format (eg, information in text or reference list)					
	3: Reference to mainly identifiable scientific studies, regardless of format (in >50% of claims)					
		**Type of study (for all articles scoring 2 or 3 on “attribution”)**			Score x 1	
			0: In vitro, animal models, and editorials			
			1: All observational works			
			2: Meta-analyses, randomized controlled trials, and clinical studies			
**Conflicts of interest**						Score x 3
	0: Endorsement or promotion of intervention designed to prevent or treat condition (eg, supplements, brain training games, and foods) within the article					
	1: Endorsement or promotion of educational products and services (eg, book and care home services)					
	2: Unbiased information					
**Currency**						Score x 1
	0: No date present					
	1: Article is dated but is 5 years or older					
	2: Article is dated within the last 5 years					
**Complimentary**						Score x 1
	0: No support of the patient-physician relationship					
	1: Support of the patient-physician relationship					
**Tone (includes title)**						Score x 3
	0: Fully supported—authors fully and unequivocally support the claims; strong vocabulary is used, such as “cure,” “guarantee,” and “easy”; use of nonconditional verb tenses mostly (eg, “can” and “will”); and no discussion of limitations					
	1: Mainly supported (authors mainly support their claims but with more cautious vocabulary, such as “can reduce your risk” or “may help prevent”, and no discussion of limitations)					
	2: Balanced or cautious support (authors’ claims are balanced by caution and include statements of limitations and contrasting findings)					

#### Readability

Web page content was assessed for readability by the general public using 4 different scales, including Gunning Fox Index (GFI), Flesch Reading Ease Scale (FRES), Flesh-Kincaid Grade Level (FKGL), and the Simple Measure of Gobbledygook (SMOG) scale (Table 2). There are many scales to measure the readability of information [46], but there is no universally accepted measurement of readability. Therefore, the combination of these 4 readability scores (ie, GFI, FRES, FKGL, and SMOG) has been used together to measure the readability of health information [33,47] in this study. Each web page URL was submitted to the Readable [48] web-based scoring tool by one researcher (DG). If the URL was directed to a PDF, the text was manually entered into the web-based generator by copying and pasting the titles and content. Text were excluded from the analysis if they were advertisements, hyperlinks, author names, or references, as these could bias the results [47]. The scores were compared to a value unique to each readability tool that indicated the content was universally readable.

**Table 2:** Tools used to measure readability, their range of scores, the score correlated to text that is readable by the general public, and the formula used to calculate the score.

Readability tool	Range	Readable by the general public	Formula
GFI^a^	0-20	<8 [49]	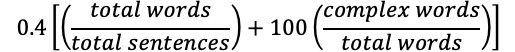
FRES^b^	0-100	>60 [47]	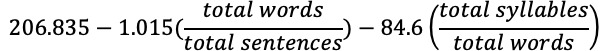
FKGL^c^	0-18	<8 [47,50]	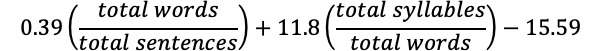
SMOG^d^	—^e^	<10 [47]	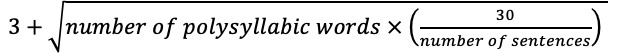

^a^GFI: Gunning Fox Index.

^b^FRES: Flesch Reading Ease Scale.

^c^FKGL: Flesch-Kincaid Grade Level.

^d^SMOG: Simple Measure of Gobbledygook.

^e^Not applicable.

#### Accuracy

The 5Cs Website Evaluation Tool is a structured tool that evaluates websites using 36 questions, grouped into the following 5 accuracy criteria: credibility, currency, content, construction, and clarity [51]. Since the construction, credibility, currency, and content of websites included in this study were assessed with the quality and readability tools, we only applied the content criteria.

The tool asks if the information on the website is evidence based and represents information from published journals and books [51]. To complete this assessment, current evidence from peer-reviewed journals was gathered, as they pertain to 5 key topics related to cannabis and driving (Multimedia Appendix 1). These topics include (1) the effects of cannabis consumption, (2) the prevalence of DUIC, (3) the effects of cannabis on driving performance, (4) risk of collision after using cannabis, and (5) the detection of cannabis-impaired drivers by law enforcement.

Each web page was assessed for the content across the 5 key categories. For each topic, the web page content was rated as accurate, not accurate, mixed accuracy (ie, some statements were accurate and some were not, or information was not aligning with the literature), or information not present. Each web page was rated independently by 2 researchers (MJ and DG); discrepancies were discussed and resolved. Web pages categorized as peer-reviewed (ie, peer-reviewed journal articles) were not included in the accuracy analysis, as peer-reviewed literature was used to create the evidence-based summary used in the content assessment. This approach has been used by others conducting similar content analyses [21].

### Statistical Analysis

Descriptive statistics was performed with the mean (μ), standard deviation (σ), and total sample size (n) being reported for the average QUEST score of all web pages and by category. To assess correlations between QUEST scores and readability scores (ie, GFI, FRES, FKGL, and SMOG), a Pearson 2-tailed test was performed [33]. To assess the QUEST score for the presence of the HON code, an unpaired 1-tailed *t* test was performed, testing if the HON code was present on web pages with higher QUEST scores [33].

### Ethics Approval

This study was exempted from ethical approval because it does not involve human participants.

## Results

### Overview

**Figure 1:**
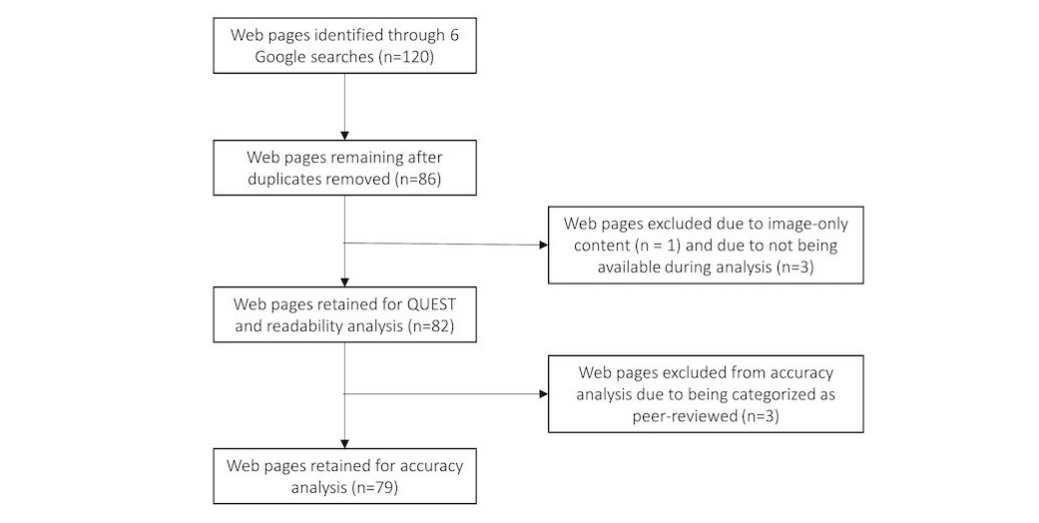
Included and excluded web pages for quantitative analysis of quality, readability, and accuracy. QUEST: quality evaluation scoring tool.

A total of 120 web pages were identified for analysis (Multimedia Appendix 2). Of the 120 web pages, 34 were removed as duplicate web pages, and 4 were removed as they did not meet the eligibility criteria, leaving 82 web pages included in the study (Figure 1). Of these, 40% (33/82) of web pages were categorized as digital media, 20% (16/82) as commercial web pages, 13% (11/82) as government organizations, 12% (10/82) as health organizations, 10% (8/82) as nonprofit foundations, 4% (3/82) as peer-reviewed content, and 1% (1/82) as “other.” Multimedia Appendix 3 presents the web pages included in the data analysis.

### Quality

The range of the QUEST scores was between 7 and 27, with the average Quest score being 17.4 (SD 5.6) out of a total of 28 (Table 3). Average QUEST scores by category showed that the peer-reviewed category had the highest quality with a score of 26.3 out of 28, and government web pages scored the lowest at 10.0/28.

The HON code was only present on 4 (5%) web pages, and they were found in the categories labelled as commercial (n=2), nonprofit (n=1), or health organization (n=1). There was no significant difference (*P*=.2) between the presence of a HON code on a website and the QUEST score without a HON code. Multimedia Appendix 4 presents the data from the full quality evaluation for each web page.

**Table 3:** Quality Evaluation Scoring Tool (QUEST) scores by category.

Category	QUEST score		
	Mean (μ)	SD (σ)	Total, n
Peer-reviewed content	26.3	0.6	3
Health organizations	20.5	5.6	10
Other	N/A^a^	N/A	1
Digital media	19.5	3.5	33
Commercial	15.7	6.0	16
Nonprofit foundations	14.9	4.2	8
Government	10.0	2.5	11
Total	18.1	5.6	82

^a^N/A: not applicable.

### Readability

The average readability scores were 9.7 (SD 2.3) for FKGL, 11.4 (SD 2.8) for GFI, 12.2 (SD 1.9) for SMOG index, and 49.9 (SD 12.3) for FRES. Assessing the readability scores for all web pages in relation to the universal readability score, 19.5% (16/82) of the web pages were universally readable by the FKGL score (score <8 considered universally readable), 16% (13/82) by the FRES score (score >60 considered universally readable), 11.1% (9/82) by the SMOG index (score <10 considered universally readable), and 9.8% (8/82) by the GFI score (score <8 considered universally readable). None of the web pages in the peer-reviewed or other categories were considered universally readable by any readability scoring tool (Table 4). Multimedia Appendix 5 presents the readability scores for each web page.

**Table 4:** Web pages by category that were considered universally readable.

Category	Web pages, n (%)			
	FKGL^a^	GFI^b^	SMOG^c^	FRES^d^
Commercial (n=16)	3 (19)	2 (13)	1 (6)	4 (25)
Digital media (n=33)	4 (12)	2 (6)	3 (9)	3 (9)
Government (n=11)	4 (36)	3 (27)	3 (27)	4 (36)
Other (n=1)	0 (0)	0 (0)	0 (0)	0 (0)
Nonprofit foundations (n=8)	2 (25)	0 (0)	1 (13)	1 (13)
Peer-reviewed material (n=3)	0 (0)	0 (0)	0 (0)	0 (0)
Health organization (n=10)	3 (30)	1 (10)	1 (10)	1 (10)

^a^FKGL: Flesh-Kincaid Grade Level.

^b^GFI: Gunning Fox Index.

^c^SMOG: Simple Measure of Gobbledygook.

^d^FRES: Flesch Reading Ease Scale.

### Correlation Between Quality and Readability

A Pearson 2-tailed test showed a significant positive correlation between the QUEST score and the FKGL (*r*=0.41; *P*<.001), GFI (*r*=0.28; *P*=.01), and SMOG (*r*=0.34; *P*=.002) readability scores. A negative correlation was found between the QUEST score and the FRES score (*r*=–0.40; *P*<.001).

### Accuracy

**Figure 2:**
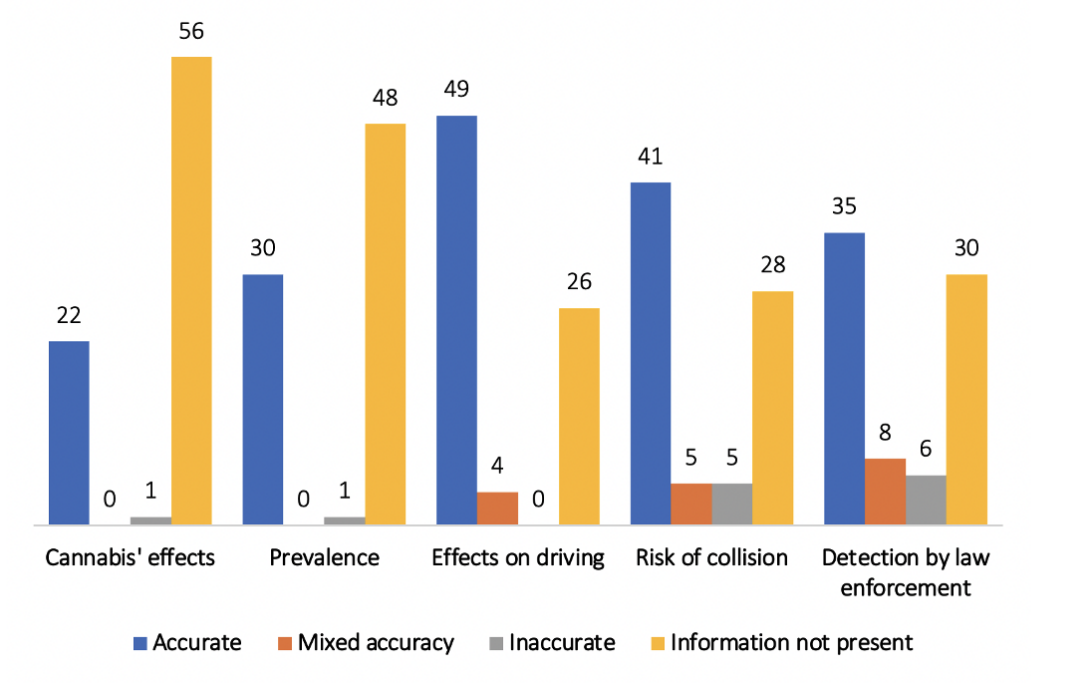
Accuracy ratings of web page content. Content accuracy of the 5 key topics about driving under the influence of cannabis are represented with colors.

Of the 79 web pages that were eligible to be reviewed for accuracy, 23 web pages discussed information related to the timing of the effects from cannabis consumption; 31 web pages were related to the prevalence of DUIC; 53 were related to the effects of cannabis impairment on driving ability; 51 were related to the risk of collision; and 49 had information related to detection by law (Figure 2). From those, 96% (22/23) had accurate information on the effects from cannabis consumption; 97% (30/31) of the web pages had accurate information about the prevalence of DUIC; 92% (49/53) of the web pages presented accurate information on the effects of cannabis impairment on driving ability; 80% (41/51) of the web pages had accurate information on the risk of collision; and 71% (35/49) of the web pages presented accurate information on detection by law. Sample excerpts from web pages and accuracy categorization are included in Multimedia Appendix 6.

## Discussion

### Principal Findings

This study assessed the quality, readability, and accuracy of information about DUIC found on the internet using the Google search engine. Our findings showed that peer-reviewed papers had the highest quality of information; however, these web pages were not considered universally readable. The difficulty with comprehension may lead to misinterpretation and inaccurate expectations [52,53].

Surprisingly, our research indicated that government web pages were rated as having the lowest quality, contrary to the general perception that government sources would contain high-quality information. This low rating was likely attributed to the tone of the presented text, given much of the information on government websites was one-sided, used strong words such as “will,” and did not discuss limitations of the information presented. This low quality could be due to the fact that government websites often presented information on laws and regulations and did not provide references to other information. This is unfortunate, as government web pages are typically viewed as an accurate source of information as indicated by various academic guides for evaluating information sources [54,55].

Readability for the public was problematic for most pages, with less than 20% of all web pages considered readable based on the FKGL, GFI, SMOG, and FRES readability tools. The majority of the content was written at a higher level of reading, which would often be used in academic settings or postsecondary education. The paucity of web pages written at levels that were considered universally readable was consistent with other health information topics on the internet (eg, general surgical procedures [56] and total joint arthroplasty [57]), suggesting that this could be a wider issue than solely information on cannabis [33,47,58]. Kruger et al [58] also suggests that significant efforts are still needed to provide accurate cannabis-related information on the internet for the health and safety of individuals and society [58].

The readability could be contributing to the misperceptions and behaviors; however, further studies assessing the interpretation of high-quality information with low readability scores could be beneficial. Associations and health advocacy groups should consider the health literacy of the public [59] when creating content to educate the public on DUIC. In addition, a more active form of education for the public could be beneficial as opposed to the passive information presented on a web page.

Our research shows that 80% of the information available about DUIC and its risks for accidents was accurate. However, although most information on DUIC was accurate, it was the lack of complete information that was most concerning. Of the 79 web pages that were analyzed for information about DUIC, 48% (n=38) either had no information on the risk of collision or had mixed or inaccurate information. Misperceptions surrounding cannabis particularly do not recognize the increased risk of accidents associated with DUIC, which highlights the need for comprehensive and accurate information [6,10], as many people turn to the internet to find information about cannabis [8,60] and about health in general [61].

Contrary to our finding that many web pages generally presented accurate information regarding DUIC, Lau et al [20] found that around 80% of the internet claims were inaccurate when investigating the information related to cannabis health benefits. This may suggest that the evidence regarding DUIC is less debated compared to suggested health benefits of cannabis; still, DUIC behaviors persist despite the presence of easily accessible accurate information [5,6]. Studies have shown that both adolescents and adults have a low risk perception of cannabis [62] and feel they are in control of their driving after cannabis consumption [63]. This is problematic given the evidence that cannabis can significantly impair motor coordination, judgment, and reaction time [64,65], increasing the risk of motor vehicle accidents [66].

### Limitations

This study has a number of limitations. Although we have used what appears to be the most appropriate tools to evaluate web page information, there are no best practices for conducting this type of research. Second, the QUEST tool does not have a target quality score or a threshold of acceptable quality, and therefore, we can only make relative comparisons with the web pages included in this study. Finally, we made the assumption that peer-reviewed content was accurate and excluded those sites from the accuracy assessment. However, there is no guarantee that all peer-reviewed materials are fully accurate. Fortunately, only 3 web pages fell into this category, so this would have minimal impact on the overall analysis.

### Conclusions

Most of the identified web pages on Google Canada search engine provided accurate information about DUIC; however, the information was incomplete, the readability was generally low, and the quality of information varied depending on the source. Health organizations should consider health literacy of the public when creating content to help prevent misinterpretation and perpetuate prevailing misperceptions surrounding DUIC. Delivering high-quality, readable, and accurate information in a way that is comprehensible to the public is needed to support informed decision-making.

## Appendix

Multimedia Appendix 1 Supplementary material.

Multimedia Appendix 2 Web pages collected form each Google page.

Multimedia Appendix 3 Web pages included in the data analysis.

Multimedia Appendix 4 The quality assessment of the included web pages.

Multimedia Appendix 5 The readability scores of the included web pages.

Multimedia Appendix 6 Examples of quotations used for the accuracy assessment.

## Abbreviations

DUIC: driving under the influence of cannabis
FKGL: Flesch-Kincaid Grade Level
FRES: Flesch Reading Ease Scale
GFI: Gunning Fox Index
HON: Health on the Net
QUEST: quality evaluation scoring tool
SMOG: Simple Measure of Gobbledygook
