# Supplementary material for: The Quality, Readability, and Accuracy of the Information on Google About Cannabis and Driving: Quantitative Content Analysis
Source: JMIR Infodemiology. 2023 May 2;3:e43001. doi: 10.2196/43001 (PMC10189625; doi:10.2196/43001)
Supplement: Multimedia Appendix 1 [file infodemiology_v3i1e43001_app1.pdf]

Multimedia Appendix 1

Supplementary material

Evidence on five key topics about Cannabis and Driving

Evidence of the effects of cannabis consumption

Cannabis consumption, THC concentration, body fat, and experience with cannabis all affect THC blood levels; regular cannabis users can develop some tolerance to cannabis impairment effects; the route of administration determines how fast the effect begins and how long it lasts (smoke/vape is quicker to take effect, less duration; oral is slower to take effect, longer duration)

- THC blood levels depend on the amount ingested or smoked, the concentration of THC in cannabis, the amount of body fat, and the extent of experience with cannabis [1]
- Smoking/vaporizing: onset in 5-10 min; duration of effect 2-4 hours; peak 10-30 mins [2]
- Oral: onset in 1- 3 hours; duration of effect 6-8 hours; peak 2-4 hours [2]
- Evidence suggests that regular cannabis users can develop tolerance to the impairment effects of cannabis [3]

Prevalence of Cannabis Use and Driving

The practice of driving after cannabis use is common; it is also common for people to ride as passengers in vehicles driven by cannabis users.

- In the past 12 months, 15% of individuals with a valid driver's license who use cannabis admitted driving within 2 hours of using cannabis [4].
- Cannabis use and driving were more prevalent among men than among women [4].
- Passengers are commonly found riding in a vehicle driven by someone who has used cannabis [5]
- The prevalence of cannabis use among nighttime drivers is higher than that of alcohol [6].
- Many young Canadians do not necessarily perceive their driving ability as adversely affected by cannabis use; this may contribute to the prevalence of driving after cannabis use [6].

Effects of Cannabis on Driving Performances

Cognitive tasks, such as tracking, reaction time, visual function, concentration, short-term memory, response time, and divided attention, are negatively affected by cannabis, depending on the amount, strength, experience with cannabis, frequency, and experience of the driver; the impact is magnified by the combination of cannabis with alcohol or other drugs.

- A driver's ability to operate a vehicle safely is also adversely affected by cannabis use [7].
- It is likely that the extent of the adverse effects of cannabis use will depend on the amount of cannabis used, the strength, experience with cannabis, and frequency and experience of the driver [1].
- It is associated with performance deficits in tracking, reaction time, visual function, concentration, short-term memory, and divided attention [7-9].
- Increased variability in lateral position in the lane [8]
- Impaired performance on divided attention tasks [9]
- Produce greater variability in steering wheel adjustments [1]
- In addition to greater variability in speed and following distance, cannabis-using drivers have a slower reaction time when sudden changes occur in the environment [1]
- As a result of overestimating the degree of impairment due to cannabis, drivers may try to compensate by driving slower and leaving longer following distances [1]
- As driving demands increase, impairment becomes increasingly evident [1]
- Driving skills were negatively affected by cannabis and alcohol when combined [8]

Risk of Collision after Using Cannabis

Combined with alcohol and at high doses, cannabis can increase the risk of a crash; cannabis is the most common psychoactive substance found in deceased and injured drivers in Canada; cannabis-related fatalities and injuries are more prevalent among 16-34-year-olds.

- Among drivers involved in serious accidents, cannabis is frequently found [6].
- The risk of collisions has not increased significantly in some studies [10]
- Researchers have also shown that low levels of cannabis use increase crash risk, and crash risk increases with dose [11-13].
- According to meta-analytic research, cannabis doubles the chances of being involved in a crash [14].

- There is a significantly increased risk of collision for drivers who consume cannabis and alcohol at the same time [11,15,16].
- In Canada, cannabis is among the most commonly found psychoactive substances among dead and injured drivers [17].
- In Canada, 16-34-year-olds make up only 32% of the population, but 61% of cannabis-related deaths [18].
- In addition, young adults aged 16 - 34 made up 59% of the cannabis-attributable injuries, as well as 68% of the cannabis-attributable property damage only (PDO) collisions [18].

Detection of Cannabis use by Impaired Drivers

Trained officers can accurately detect impairment by cannabis.

- Young people believe it is difficult for law enforcement officers to detect and charge drivers who are under the influence of cannabis [19,20].
- An officer trained in drug identification has a 95% accuracy rate in identifying the type of drug causing impairment [21].

References

1. Canadian Centre on Substance Abuse. Cannabis, driving and implications for youth. Canada Commons. 2016. http://www.deslibris.ca/ID/248544 [accessed Dec 21, 2021]
2. *Cannabis 101: Burning Questions About THC, CBD and Everything in Between*. 2021. https://www.youtube.com/watch?v=LvqNwFmfCIo [accessed 21, Dec 2021]
3. Readfield GM, Goldberger BA, Gold MS, DuPont RL. The mirage of impairing drug concentration thresholds: A rationale for zero tolerance per se driving under the influence of drugs laws. J Anal Toxicol. 2012;*36*(5):353–356. https://doi.org/10.1093/jat/bks037
4. The Daily. National Cannabis Survey, first quarter 2019. Statistics Canada. May 2 2019. https://www150.statcan.gc.ca/n1/daily-quotidien/190502/dq190502a-eng.htm [accessed Dec 21, 2021]
5. Beirness DJ. The characteristics of youth passengers of impaired drivers. Canadian Centre on Substance Abuse. 2014 https://www.ccsa.ca/sites/default/files/2019-05/CCSA-Characteristics-Youth-Passengers-Impaired-Drivers-technical-report-2014-en.pdf [accessed Dec 21, 2021]
6. Beirness DJ, Porath AJ. Cannabis Use and Driving – An Update. CCSA. 2019. https://www.ccsa.ca/sites/default/files/2019-10/CCSA-Cannabis-Use-Driving-Report-2019-en_1.pdf [accessed Dec 21, 2021].
7. Hartman RL, Huestis MA. Cannabis effects on driving skills. Clin Chem. 2013;*59*(3):478–492. https://doi.org/10.1373/clinchem.2012.194381
8. Hartman RL, Brown TL, Milavetz G, Spurgin A, Pierce RS, Gorelick DA, Gaffney G, Huestis MA. Cannabis effects on driving lateral control with and without alcohol. Drug Alcohol Depend. 2015;*154*:25–37. https://doi.org/10.1016/j.drugalcdep.2015.06.015
9. Pearlson GD, Stevens MC, D'Souza DC. Cannabis and Driving. Front Psychiatry. 2021;12. https://doi.org/10.3389/fpsyt.2021.689444

10. Lacey JH, Kelley-Baker T, Berning A, Romano E, Ramirez A, Yao J, Moore C, Brainard K, Carr K, Pell K, Compton R. Drug and Alcohol Crash Risk: A Case-Control Study (Report No. DOT HS 812 355). National Highway Traffic Safety Administration. 2016. https://one.nhtsa.gov/staticfiles/nti/impaired_driving/pdf/812355_DrugAlcoholCrashRisk.pdf [accessed Dec 21, 2021]

11. Drummer OH, Gerostamoulos J, Batziris H, Chu M, Caplehorn J, Robertson MD, Swann P. The involvement of drugs in drivers of motor vehicles killed in Australian road traffic crashes. Accid Anal Prev. 2004;*36*(2):239–248. https://doi.org/10.1016/s0001-4575(02)00153-7

12. Laumon B, Gadegbeku B, Martin JL, Biecheler MB, SAM Group. Cannabis intoxication and fatal road crashes in France: Population based case-control study. BMJ*. 2005;331*(7529):1371. https://doi.org/10.1136/bmj.38648.617986.1F

13. Mura P, Kintz P, Ludes B, Gaulier JM, Marquet P, Martin-Dupont S, Vincent F, Kaddour A, Goullé JP, Nouveau J, Moulsma M, Tilhet-Coartet S, Pourrat O. Comparison of the prevalence of alcohol, cannabis and other drugs between 900 injured drivers and 900 control subjects: Results of a French collaborative study. Forensic Sci Int. 2003;133(1–2):79–85. https://doi.org/10.1016/s0379-0738(03)00052-5

14. Asbridge M, Hayden JA, Cartwright JL. Acute cannabis consumption and motor vehicle collision risk: Systematic review of observational studies and meta-analysis. BMJ. 2012;344:e536. https://doi.org/10.1136/bmj.e536

15. Dussault C, Brault M, Bouchard J, Lemire A. The Contribution of Alcohol and Other Drugs Among Fatally Injured Drivers in Quebec: Some Preliminary Results. 2004. https://www.semanticscholar.org/paper/THE-CONTRIBUTION-OF-ALCOHOL-AND-OTHER-DRUGS-AMONG-Dussault-Brault/a8735eecf1854c667df318db8543e16a16f15538 [accessed Dec 21, 2021]

16. Longo MC, Hunter CE, Lokan RJ, White JM, White MA. The prevalence of alcohol, cannabinoids, benzodiazepines and stimulants amongst injured drivers and their role in driver culpability: Part II: the relationship between drug prevalence and drug concentration, and driver culpability. Accid Anal Prev. 2000;*32*(5):623–632. https://doi.org/10.1016/s0001-4575(99)00110-4
17. Beasley E, Beirness D. Drug Use by Fatally Injured Drivers in Canada (2000—2008). CCSA. 2011. http://eggsonweed.ca/wp-content/uploads/2016/11/Drug_Use_by_Fatally_Injured_Drivers_in_Canada.pdf [accessed Dec 21, 2021]
18. Wettlaufer A, Florica RO, Asbridge M, Beirness D, Brubacher J, Callaghan R, Fischer B, Gmel G, Imtiaz S, Mann RE, McKiernan A, Rehm J. Estimating the harms and costs of cannabis-attributable collisions in the Canadian provinces. Drug Alcohol Depend. 2017;*173*:185–190. https://doi.org/10.1016/j.drugalcdep.2016.12.024
19. McKiernan A, Fleming K. Canadian Youth Perceptions on Cannabis. Canada Commons. 2017. http://www.deslibris.ca/ID/10065775 [accessed Dec 21, 2021]
20. Porath-Waller AJ, Brown JE, Frigon AP, Clark H. *What Canadian youth think about cannabis: Technical report*. Canada Commons. 2014. https://www.deslibris.ca/ID/240336 [accessed Dec 21, 2021]
21. Beirness D, Beasley E, Lecavalier J. The Accuracy of Evaluations by Drug Recognition Experts in Canada. J Can Soc Forensic Sci. 2009;*42*:75–79. https://doi.org/10.1080/00085030.2009.10757598
