# Supplementary material for: The Quality, Readability, and Accuracy of the Information on Google About Cannabis and Driving: Quantitative Content Analysis
Source: JMIR Infodemiology. 2023 May 2;3:e43001. doi: 10.2196/43001 (PMC10189625; doi:10.2196/43001)
Supplement: Multimedia Appendix 2 [file infodemiology_v3i1e43001_app2.pdf]

Multimedia Appendix 2

Table S1 : All web pages collected from each Google search

Note: The search terms used and the category of the webpage before eligibility criteria was met. Those marked with '-' under web page category were the articles that were excluded from analysis.

	Web Page	Search Term	Web page category
1	https://canadasafetycouncil.org/how-cannabis-use-affects-driving/?gclid=EAIaIQobChMIsO65g5r48wIVQeWzCh3LTgqzEAAYASAAEgKOhPD_BwE	Cannabis and driving	Non-profit
2	https://madd.ca/pages/impaired-driving/overview/cannabis-and-driving/?gclid=EAIaIQobChMIsO65g5r48wIVQeWzCh3LTgqzEAAYAiAAEgL9j_D_BwE	Cannabis and driving	Non-profit
3	https://www.frontiersin.org/articles/10.3389/fpsyt.2021.689444/full	Cannabis and driving	Peer reviewed
4	https://www.ontario.ca/page/cannabis-and-driving	Cannabis and driving	Government
5	https://www.heretohelp.bc.ca/infosheet/cannabis-and-driving	Cannabis and driving	Health Organization
6	https://www.canada.ca/en/services/policing/police/community-safety-policing/impaired-driving/drug-impaired-driving.html	Cannabis and driving	Government
7	https://www.drugabuse.gov/publications/research-reports/marijuana/does-marijuana-use-affect-driving	Cannabis and driving	Health Organization
8	https://www.canadadrives.ca/blog/driving-tips/cannabis-impaired-driving-dui-laws-canada	Cannabis and driving	Commercial
9	https://www.sgi.sk.ca/cannabis-use-and-driving	Cannabis and driving	Government
10	https://globalnews.ca/news/6245267/cannabis-and-driving-study-thc/	Cannabis and driving	Digital media
11	https://novascotia.ca/cannabis/impaired-driving/	Cannabis and driving	Government
12	https://www.caa.ca/driving-safely/cannabis-impaired-driving/	Cannabis and driving	Commercial
13	https://tirf.ca/projects/status-of-alcohol-impaired-driving-in-canada/?gclid=EAIaIQobChMI2cbMqpz48wIV-AaICR2_tAvkEAAYASAAEgJtlPD_BwE	Cannabis and driving	Non-profit
14	https://www.edmontonpolice.ca/TrafficVehicles/ImpairedDriving/Cannabis	Cannabis and driving	Government

15	https://www.forbes.com/sites/chrisroberts/2021/06/29/study-marijuanas-impact-on-driving-is-strain-specific/	Cannabis and driving	Digital media
16	https://www.alberta.ca/cannabis-and-driving.aspx	Cannabis and driving	Government
17	https://www.ccsa.ca/sites/default/files/2019-04/CCSA-Cannabis-Use-Driving-Report-2017-en.pdf	Cannabis and driving	-
18	https://www.saskatchewan.ca/government/cannabis-in-saskatchewan/cannabis-and-driving	Cannabis and driving	Government
19	https://aglc.ca/cannabis/using-cannabis-responsibly/cannabis-and-impaired-driving	Cannabis and driving	-
20	https://en.wikipedia.org/wiki/Cannabis_and_impaired_driving	Cannabis and driving	Other
21	https://www.drugabuse.gov/publications/research-reports/marijuana/does-marijuana-use-affect-driving	Marijuana and driving	Health Organization
22	https://www.canada.ca/en/services/policing/police/community-safety-policing/impaired-driving/drug-impaired-driving.html	Marijuana and driving	Government
23	https://www.canadadrives.ca/blog/driving-tips/cannabis-impaired-driving-dui-laws-canada	Marijuana and driving	Commercial
24	https://www.forbes.com/sites/chrisroberts/2021/06/29/study-marijuanas-impact-on-driving-is-strain-specific/	Marijuana and driving	Digital media
25	https://madd.ca/pages/impaired-driving/overview/cannabis-and-driving/	Marijuana and driving	Non-profit
26	https://www.verywellmind.com/how-does-marijuana-affect-driving-63533	Marijuana and driving	Health Organization
27	https://www.iii.org/article/background-on-marijuana-and-impaired-driving	Marijuana and driving	Commercial
28	https://www.ncbi.nlm.nih.gov/pmc/articles/PMC2722956/	Marijuana and driving	Peer reviewed
29	https://drugpolicy.org/does-marijuana-impair-driving-way-alcohol-does	Marijuana and driving	Non-profit
30	https://www.cdc.gov/marijuana/pdf/marijuana-driving-508.pdf	Marijuana and driving	-
31	https://www.theguardian.com/society/2019/apr/26/driving-while-high-cannabis-study-safety	Marijuana and driving	Digital media
32	https://www.aappublications.org/news/2020/01/24/parentplus012420	Marijuana and driving	Health Organization
33	https://www.ncsl.org/research/transportation/drugged-driving-overview.aspx	Marijuana and driving	Digital media
34	https://en.wikipedia.org/wiki/Cannabis_and_impaired_driving	Marijuana and driving	Other
35	https://www.cnn.com/2020/01/14/health/weed-impact-driving-wellness/index.html	Marijuana and driving	Digital media

36	https://www.ccsa.ca/sites/default/files/2019-10/CCSA-Cannabis-Use-Driving-Report-2019-en_1.pdf	Marijuana and driving	Health Organization
37	https://www.teendriversource.org/teen-crash-risks-prevention/rules-of-the-road/impaired-driving/marijuana-use-and-driving	Marijuana and driving	Non-profit
38	https://www.insurancejournal.com/news/national/2020/01/16/555111.htm	Marijuana and driving	Digital media
39	https://www.ghsa.org/state-laws/issues/drug%20impaired%20driving	Marijuana and driving	Non-profit
40	https://medicalmarijuana.ca/resource-center/driving-cannabis/	Marijuana and driving	Commercial
41	https://madd.ca/pages/impaired-driving/overview/cannabis-and-driving/	Weed and driving	Health Organization
42	https://www.canadadrives.ca/blog/driving-tips/cannabis-impaired-driving-dui-laws-canada	Weed and driving	Commercial
43	https://www.theguardian.com/society/2019/apr/26/driving-while-high-cannabis-study-safety	Weed and driving	Digital media
44	https://www.vice.com/en/article/g5pk34/is-it-safe-to-smoke-cannabis-and-drive	Weed and driving	Digital media
45	https://www.verywellmind.com/how-does-marijuana-affect-driving-63533	Weed and driving	Health Organization
46	https://en.wikipedia.org/wiki/Cannabis_and_impaired_driving	Weed and driving	Other
47	https://www.cnn.com/2020/01/14/health/weed-impact-driving-wellness/index.html	Weed and driving	Digital media
48	https://www.insurancejournal.com/news/national/2020/01/16/555111.htm	Weed and driving	Commercial
49	https://www.shutterstock.com/search/weed+driving	Weed and driving	-
50	https://news.westernu.ca/2021/06/the-whys-of-weed-behind-the-wheel/	Weed and driving	Digital media
51	https://www.nbcnews.com/health/health-news/smoking-weed-when-someone-too-high-drive-n954211	Weed and driving	Digital media
52	https://www.thinkinsure.ca/insurance-help-centre/driving-high-in-ontario.html	Weed and driving	Commercial
53	https://www.qredible.co.uk/b/driving-under-influence-cannabis/	Weed and driving	Commercial
54	https://www.theglobeandmail.com/globe-drive/culture/commuting/how-much-marijuana-can-i-have-and-still-be-safe-to-drive/article27897258/	Weed and driving	Digital media
55	https://pubmed.ncbi.nlm.nih.gov/23300977/	Weed and driving	Peer reviewed
56	https://www.westword.com/marijuana/how-much-weed-can-be-smoke-before-legally-driving-11803572	Weed and driving	Digital media

57	https://www.caaquebec.com/en/caa-quebec-foundation/current-issues/marijuana-and-driving/	Weed and driving	Commercial
58	https://www.majlaw.co.uk/offences/drug-driving/cannabis/	Weed and driving	Commercial
59	https://www.caa.ca/driving-safely/cannabis-impaired-driving/	Weed and driving	Commercial
60	https://www.caranddriver.com/features/a15141572/puff-the-dangerous-driver-is-doping-and-driving-safe-archived-feature/	Weed and driving	Digital media
61	https://www.cpha.ca/pot-driving	Pot and driving	Health Organization
62	https://www.canadadrives.ca/blog/driving-tips/cannabis-impaired-driving-dui-laws-canada	Pot and driving	Commercial
63	https://www.theguardian.com/society/2019/apr/26/driving-while-high-cannabis-study-safety	Pot and driving	Digital media
64	https://www.caa.ca/app/uploads/2021/02/CPHA-Pot-Driving-FAQ-EN.pdf	Pot and driving	Commercial
65	https://www.cbc.ca/news/canada/kitchener-waterloo/driving-with-pot-in-the-vehicle-here-s-what-opp-say-you-need-to-know-1.4930345	Pot and driving	Digital media
66	https://www.cbc.ca/news/canada/windsor/madd-pot-driving-1.4865890	Pot and driving	Digital media
67	https://driving.ca/column/lorraine/legally-determining-impairment-from-pot-not-as-clear-as-we-thought	Pot and driving	Digital media
68	https://www.webmd.com/mental-health/addiction/news/20190425/many-driving-on-pot-even-with-kids-in-car	Pot and driving	Commercial
69	https://globalnews.ca/news/5386321/cananbis-crash-risk/	Pot and driving	Digital media
70	https://www.bnnbloomberg.ca/pot-users-know-driving-high-is-bad-but-many-do-it-anyway-health-canada-1.1171322	Pot and driving	Digital media
71	https://www.thinkinsure.ca/insurance-help-centre/driving-high-in-ontario.html	Pot and driving	Commercial
72	https://torontosun.com/news/provincial/too-early-to-determine-whether-pot-impaired-driving-on-the-increase	Pot and driving	Digital media
73	https://nationalpost.com/news/thinking-of-driving-while-high-a-new-study-shows-pot-increases-the-risk-of-a-crash-even-five-hours-after-inhaling	Pot and driving	Digital media
74	https://www.usnews.com/news/health-news/articles/2020-12-30/with-pot-rules-relaxed-more-us-teens-driving-while-high-study	Pot and driving	Digital media
75	https://pacificlaw.ca/is-it-legal-to-smoke-pot-and-drive/	Pot and driving	Commercial

76	https://en.wikipedia.org/wiki/Cannabis_and_impaired_driving	Pot and driving	Other
77	https://www.healthline.com/health-news/cannabis-use-can-affect-your-driving-long-after-your-high-is-gone	Pot and driving	Commercial
78	https://ruor.uottawa.ca/bitstream/10393/30262/1/factsheet_e.pdf	Pot and driving	Health Organization
79	https://www.theglobeandmail.com/drive/mobility/article-how-long-should-i-be-waiting-to-drive-after-consuming-edible-cannabis/	Pot and driving	Digital media
80	https://www.ncbi.nlm.nih.gov/pmc/articles/PMC4361101/	Pot and driving	Digital media
81	https://www.canada.ca/en/campaign/don-t-drive-high.html	Driving high	Government
82	https://www.canadadrives.ca/blog/driving-tips/cannabis-impaired-driving-dui-laws-canada	Driving high	Commercial
83	https://www.theguardian.com/society/2019/jul/21/how-dangerous-is-stoned-driving	Driving high	Digital media
84	https://madd.ca/pages/impaired-driving/overview/cannabis-and-driving/	Driving high	Health Organization
85	https://www.nytimes.com/2022/04/12/well/live/driving-while-high-marijuana.html	Driving high	Digital media
86	https://www.surex.com/blog/driving-high	Driving high	Commercial
87	https://www.codot.gov/safety/impaired-driving/druggeddriving	Driving high	Government
88	https://www.med.ubc.ca/news/more-canadians-may-be-driving-high-since-cannabis-legalization/	Driving high	Health Organization
89	https://www.justthefactspei.ca/driving	Driving high	Government
90	https://www.caa.ca/driving-safely/cannabis-impaired-driving/	Driving high	Commercial
91	https://www.healthychildren.org/English/ages-stages/teen/substance-abuse/Pages/Driving-While-High-a-Dangerous-Mix.aspx	Driving high	Non-profit
92	https://www.drugfreekidscanada.org/prevention/issues/drugs-and-driving/	Driving high	Non-profit
93	https://ama.ab.ca/community/build/risky-driving/impaired-driving/cannabis-dui	Driving high	Commercial
94	https://www.sgi.sk.ca/cannabis-use-and-driving	Driving high	Government
95	https://www.verywellmind.com/how-does-marijuana-affect-driving-63533	Driving high	Health Organization
96	https://www.thinkinsure.ca/insurance-help-centre/driving-high-in-ontario.html	Driving high	Commercial
97	https://nida.nih.gov/publications/research-reports/marijuana/does-marijuana-use-affect-driving	Driving high	Health Organization

98	https://explore.ucalgary.ca/does-cannabis-impair-driving	Driving high	Health Organization
99	https://globalnews.ca/news/6340319/young-canadians-driving-cannabis/	Driving high	Digital media
100	https://www.turnbridge.com/news-events/latest-articles/driving-while-high/	Driving high	Commercial
101	https://www.canadadrives.ca/blog/driving-tips/cannabis-impaired-driving-dui-laws-canada	Driving Stoned	Commercial
102	https://www.theguardian.com/society/2019/jul/21/how-dangerous-is-stoned-driving	Driving stoned	Digital media
103	https://www.canada.ca/en/services/policing/police/community-safety-policing/impaired-driving/drug-impaired-driving.html	Driving stoned	Government
104	https://www.forbes.com/sites/chrisroberts/2021/06/29/study-marijuanas-impact-on-driving-is-strain-specific/	Driving stoned	Digital media
105	https://madd.ca/pages/impaired-driving/overview/cannabis-and-driving/	Driving stoned	Health Organization
106	https://www.verywellmind.com/how-does-marijuana-affect-driving-63533	Driving stoned	Health Organization
107	https://www.wired.com/story/weed-dui-test/	Driving stoned	Digital media
108	https://www.cbsnews.com/news/driving-while-high-offenders-dont-think-theyll-be-arrested/	Driving stoned	Digital media
109	https://fherehab.com/learning/the-dangers-of-driving-stoned/	Driving stoned	Commercial
110	https://consumer.healthday.com/1-31-people-high-on-pot-used-a-driving-simulator-here-s-what-happened-2656490096.html	Driving stoned	Digital media
111	https://www.vice.com/en/article/g5pk34/is-it-safe-to-smoke-cannabis-and-drive	Driving stoned	Digital media
112	https://time.com/3706491/driving-stoned-drunk-study-safety/	Driving stoned	Digital media
113	https://www.sgi.sk.ca/news?title=think-a-police-officer-can-t-tell-if-you-re-driving-stoned--think-again-	Driving stoned	Government
114	https://www.usnews.com/news/health-news/articles/2022-02-01/people-high-on-pot-used-a-driving-simulator-heres-what-happened	Driving stoned	Digital media
115	https://www.themarshallproject.org/2017/01/16/when-are-you-too-stoned-to-drive	Driving stoned	Digital media
116	https://www.justthefactspei.ca/driving	Driving stoned	Government
117	https://www.healthychildren.org/English/ages-stages/teen/substance-abuse/Pages/Driving-While-High-a-Dangerous-Mix.aspx	Driving stoned	Health Organization

118	https://globalnews.ca/news/2978925/what-happens-when-youre-caught-driving-stoned-canadas-supreme-court-may-soon-tell-us/	Driving stoned	Digital media
119	https://usa.streetsblog.org/2022/04/15/can-we-stop-stoned-driving-without-ending-car-dependency/	Driving stoned	Digital media
120	https://explore.ucalgary.ca/does-cannabis-impair-driving	Driving stoned	Health Organization
