# Supplementary material for: The Quality, Readability, and Accuracy of the Information on Google About Cannabis and Driving: Quantitative Content Analysis
Source: JMIR Infodemiology. 2023 May 2;3:e43001. doi: 10.2196/43001 (PMC10189625; doi:10.2196/43001)
Supplement: Multimedia Appendix 4 [file infodemiology_v3i1e43001_app4.pdf]

## Multimedia Appendix 4

Table S3 :The quality assessment of included web pages

| ID | HON code | QUEST TOOL | | | | | | | |
|---|---|---|---|---|---|---|---|---|---|
| | | **Authors** | **Attribution** | **Attribution2** | **Conflict of Interest** | **Date of publication** | **Complementarity** | **Tone** | **QUEST Score** |
| 1 | No | Organization takes authorship | Mention of expert source, research findings (though with insufficient information to identify the specific studies), links to various sites, advocacy body, or other | None | Unbiased information | No date present | No support of the patient-physician relationship | Mainly supported (authors mainly support their claims but with more cautious vocabulary such as 'can reduce your risk' or 'may help prevent', no discussion of limitations | 13 |
| 2 | No | Organization takes authorship | Reference to many identifiable scientific studies, regardless of format (in >50% of claims) | Meta-analyses, randomized controlled trials, clinical studies | Endorsement or promotion of educational products & services (e.g., books, care home services) | No date present | Support of the patient-physician relationship | Balanced/cautious support (authors' claims are balanced by caution, includes statements of limitations and/or contrasting findings) | 22 |
| 3 | No | Author's name and qualification clearly stated | Reference to many identifiable scientific studies, | Meta-analyses, randomized controlled trials, clinical studies | Unbiased information | Article is dated within the last 5 years | No support of the patient-physician relationship | Balanced/cautious support (authors' claims are balanced by caution, includes statements of | 27 |

| | | | | | | | | | |
|---|---|---|---|---|---|---|---|---|---|
| | | | regardless of format (in >50% of claims) | | | | | limitations and/or contrasting findings) | |
| 4 | No | Organization takes authorship | No sources | None | Unbiased information | Article is dated within the last 5 years | No support of the patient-physician relationship | Fully supported (authors fully and unequivocally support the claims, strong vocabulary such as 'cure', 'guarantee', and 'easy', mostly use of non-conditional verb tenses ('can, 'will'), no discussion of limitations) | 9 |
| 5 | No | Organization takes authorship | Reference to at least one identifiable scientific study, regardless of format (e.g. information in text, reference list) | Meta-analyses, randomized controlled trials, clinical studies | Unbiased information | No date present | No support of the patient-physician relationship | Balanced/cautious support (authors' claims are balanced by caution, includes statements of limitations and/or contrasting findings) | 21 |
| 6 | No | Organization takes authorship | Mention of expert source, research findings (though with insufficient information to identify the specific studies), links to various | All observational work | Unbiased information | Article is dated within the last 5 years | No support of the patient-physician relationship | Fully supported (authors fully and unequivocally support the claims, strong vocabulary such as 'cure', 'guarantee', and 'easy', mostly use of non-conditional verb tenses ('can, 'will'), no discussion of limitations) | 13 |

| | | | | | | | | | |
|---|---|---|---|---|---|---|---|---|---|
| | | | sites, advocacy body, or other | | | | | | |
| 7 | No | Organization takes authorship | Reference to many identifiable scientific studies, regardless of format (in >50% of claims) | Meta-analyses, randomized controlled trials, clinical studies | Unbiased information | Article is dated within the last 5 years | No support of the patient-physician relationship | Fully supported (authors fully and unequivocally support the claims, strong vocabulary such as 'cure', 'guarantee', and 'easy', mostly use of non-conditional verb tenses ('can, 'will'), no discussion of limitations) | 20 |
| 8 | No | Organization takes authorship | Mention of expert source, research findings (though with insufficient information to identify the specific studies), links to various sites, advocacy body, or other | In vitro, animal models, or editorials | Unbiased information | No date present | No support of the patient-physician relationship | Fully supported (authors fully and unequivocally support the claims, strong vocabulary such as 'cure', 'guarantee', and 'easy', mostly use of non-conditional verb tenses ('can, 'will'), no discussion of limitations) | 10 |
| 9 | No | Organization takes authorship | No sources | None | Unbiased information | Article is dated within the last 5 years | No support of the patient-physician relationship | Mainly supported (authors mainly support their claims but with more cautious vocabulary such as 'can reduce your risk' or 'may help prevent', no | 12 |

| | | | | | | | | | |
|---|---|---|---|---|---|---|---|---|---|
| | | | | | | | | discussion of limitations | |
| 10 | No | All others indications of authorship | Reference to at least one identifiable scientific study, regardless of format (e.g., information in text, reference list) | Meta-analyses, randomized controlled trials, clinical studies | Unbiased information | Article is dated within the last 5 years | No support of the patient-physician relationship | Balanced/cautious support (authors' claims are balanced by caution, includes statements of limitations and/or contrasting findings) | 23 |
| 11 | No | Organization takes authorship | Mention of expert source, research findings (though with insufficient information to identify the specific studies), links to various sites, advocacy body, or other | None | Unbiased information | No date present | No support of the patient-physician relationship | Fully supported (authors fully and unequivocally support the claims, strong vocabulary such as 'cure', 'guarantee', and 'easy', mostly use of non-conditional verb tenses ('can, 'will'), no discussion of limitations) | 10 |
| 12 | No | Organization takes authorship | Reference to at least one identifiable scientific study, regardless of format (e.g., information in text, reference list) | Meta-analyses, randomized controlled trials, clinical studies | Unbiased information | No date present | No support of the patient-physician relationship | Fully supported (authors fully and unequivocally support the claims, strong vocabulary such as 'cure', 'guarantee', and 'easy', mostly use of non-conditional verb tenses ('can, 'will'), no | 15 |

| | | | | | | | | | |
|---|---|---|---|---|---|---|---|---|---|
| | | | | | | | | discussion of limitations) | |
| 13 | No | Organization takes authorship | No sources | None | Endorsement or promotion of educational products & services (e.g., books, care home services) | No date present | No support of the patient-physician relationship | Balanced/cautious support (authors' claims are balanced by caution, includes statements of limitations and/or contrasting findings) | 10 |
| 14 | No | Organization takes authorship | Mention of expert source, research findings (though with insufficient information to identify the specific studies), links to various sites, advocacy body, or other | None | Unbiased information | No date present | No support of the patient-physician relationship | Fully supported (authors fully and unequivocally support the claims, strong vocabulary such as 'cure', 'guarantee', and 'easy', mostly use of non-conditional verb tenses ('can, 'will'), no discussion of limitations) | 10 |
| 15 | No | Author's name and qualification clearly stated | Reference to at least one identifiable scientific study, regardless of format (e.g., information in text, reference list) | All observational work | Unbiased information | Article is dated within the last 5 years | No support of the patient-physician relationship | Mainly supported (authors mainly support their claims but with more cautious vocabulary such as 'can reduce your risk' or 'may help prevent', no discussion of limitations | 20 |
| 16 | No | Organization takes authorship | Mention of expert source, research | All observational work | Unbiased information | No date present | No support of the patient-physician relationship | Mainly supported (authors mainly support their claims | 14 |

| | | | | | | | | | |
|---|---|---|---|---|---|---|---|---|---|
| | | | findings (though with insufficient information to identify the specific studies), links to various sites, advocacy body, or other | | | | | but with more cautious vocabulary such as 'can reduce your risk' or 'may help prevent', no discussion of limitations | |
| 17 | No | Organization takes authorship | No sources | None | Unbiased information | No date present | No support of the patient-physician relationship | Fully supported (authors fully and unequivocally support the claims, strong vocabulary such as 'cure', 'guarantee', and 'easy', mostly use of non-conditional verb tenses ('can, 'will'), no discussion of limitations) | 7 |
| 18 | No | No indication of authorship or username | Reference to at least one identifiable scientific study, regardless of format (e.g., information in text, reference list) | Meta-analyses, randomized controlled trials, clinical studies | Unbiased information | No date present | No support of the patient-physician relationship | Balanced/cautious support (authors' claims are balanced by caution, includes statements of limitations and/or contrasting findings) | 20 |
| 19 | Yes | Author's name and qualification clearly stated | Reference to at least one identifiable scientific | Meta-analyses, randomized controlled | Unbiased information | Article is dated within the last 5 years | Support of the patient-physician relationship | Mainly supported (authors mainly support their claims but with more | 22 |

| | | | | | | | | | |
|---|---|---|---|---|---|---|---|---|---|
| | | | study, regardless of format (e.g., information in text, reference list) | trials, clinical studies | | | | cautious vocabulary such as 'can reduce your risk' or 'may help prevent', no discussion of limitations | |
| 20 | No | Organization takes authorship | Reference to many identifiable scientific studies, regardless of format (in >50% of claims) | Meta-analyses, randomized controlled trials, clinical studies | Unbiased information | Article is dated within the last 5 years | No support of the patient-physician relationship | Balanced/cautious support (authors' claims are balanced by caution, includes statements of limitations and/or contrasting findings) | 26 |
| 21 | No | Author's name and qualification clearly stated | Reference to many identifiable scientific studies, regardless of format (in >50% of claims) | Meta-analyses, randomized controlled trials, clinical studies | Unbiased information | Article is dated, but 5 years or older | No support of the patient-physician relationship | Balanced/cautious support (authors' claims are balanced by caution, includes statements of limitations and/or contrasting findings) | 26 |
| 22 | No | Organization takes authorship | No sources | None | Unbiased information | No date present | No support of the patient-physician relationship | Mainly supported (authors mainly support their claims but with more cautious vocabulary such as 'can reduce your risk' or 'may help prevent', no discussion of limitations | 10 |

| | | | | | | | | | |
|---|---|---|---|---|---|---|---|---|---|
| 23 | No | All others indications of authorship | Reference to at least one identifiable scientific study, regardless of format (e.g., information in text, reference list) | Meta-analyses, randomized controlled trials, clinical studies | Endorsement of promotion of intervention designed to prevent to treat condition (e.g., supplements, brain training games, foods) within the article | Article is dated within the last 5 years | No support of the patient-physician relationship | Balanced/cautious support (authors' claims are balanced by caution, includes statements of limitations and/or contrasting findings) | 17 |
| 24 | No | Author's name and qualification clearly stated | No sources | None | Unbiased information | Article is dated within the last 5 years | No support of the patient-physician relationship | Fully supported (authors fully and unequivocally support the claims, strong vocabulary such as 'cure', 'guarantee', and 'easy', mostly use of non-conditional verb tenses ('can, 'will'), no discussion of limitations) | 10 |
| 25 | No | Organization takes authorship | Reference to at least one identifiable scientific study, regardless of format (e.g., information in text, reference list) | All observational work | Unbiased information | Article is dated within the last 5 years | No support of the patient-physician relationship | Balanced/cautious support (authors' claims are balanced by caution, includes statements of limitations and/or contrasting findings) | 22 |
| 26 | No | Author's name and qualification clearly stated | Mention of expert source, research findings | None | Unbiased information | Article is dated within the last 5 years | No support of the patient-physician relationship | Balanced/cautious support (authors' claims are balanced by caution, includes | 19 |

| | | | | | | | | | |
|---|---|---|---|---|---|---|---|---|---|
| | | | (though with insufficient information to identify the specific studies), links to various sites, advocacy body, or other | | | | | statements of limitations and/or contrasting findings) | |
| 27 | No | Author's name and qualification clearly stated | Reference to many identifiable scientific studies, regardless of format (in >50% of claims) | Meta-analyses, randomized controlled trials, clinical studies | Unbiased information | Article is dated, but 5 years or older | Support of the patient-physician relationship | Balanced/cautious support (authors' claims are balanced by caution, includes statements of limitations and/or contrasting findings) | 27 |
| 28 | No | Organization takes authorship | Reference to at least one identifiable scientific study, regardless of format (e.g., information in text, reference list) | All observational work | Unbiased information | No date present | No support of the patient-physician relationship | Mainly supported (authors mainly support their claims but with more cautious vocabulary such as 'can reduce your risk' or 'may help prevent', no discussion of limitations | 17 |
| 29 | No | No indication of authorship or username | Reference to at least one identifiable scientific study, regardless of format (e.g., | All observational work | Unbiased information | Article is dated within the last 5 years | No support of the patient-physician relationship | Balanced/cautious support (authors' claims are balanced by caution, includes statements of limitations and/or contrasting findings) | 21 |

| | | | | | | | | | |
|---|---|---|---|---|---|---|---|---|---|
| | | | information in text, reference list) | | | | | | |
| 30 | No | Organization takes authorship | Mention of expert source, research findings (though with insufficient information to identify the specific studies), links to various sites, advocacy body, or other | None | Unbiased information | No date present | No support of the patient-physician relationship | Mainly supported (authors mainly support their claims but with more cautious vocabulary such as 'can reduce your risk' or 'may help prevent', no discussion of limitations | 13 |
| 31 | No | Organization takes authorship | Reference to at least one identifiable scientific study, regardless of format (e.g., information in text, reference list) | Meta-analyses, randomized controlled trials, clinical studies | Unbiased information | No date present | No support of the patient-physician relationship | Balanced/cautious support (authors' claims are balanced by caution, includes statements of limitations and/or contrasting findings) | 21 |
| 32 | No | Organization takes authorship | Reference to at least one identifiable scientific study, regardless of format (e.g., information in | All observational work | Unbiased information | Article is dated within the last 5 years | No support of the patient-physician relationship | Mainly supported (authors mainly support their claims but with more cautious vocabulary such as 'can reduce your risk' or 'may help prevent', no | 19 |

| | | | | | | | | | |
|---|---|---|---|---|---|---|---|---|---|
| | | | text, reference list) | | | | | discussion of limitations | |
| 33 | No | All others indications of authorship | Reference to at least one identifiable scientific study, regardless of format (e.g., information in text, reference list) | All observational work | Unbiased information | Article is dated within the last 5 years | No support of the patient-physician relationship | Mainly supported (authors mainly support their claims but with more cautious vocabulary such as 'can reduce your risk' or 'may help prevent', no discussion of limitations | 19 |
| 34 | No | Organization takes authorship | Reference to at least one identifiable scientific study, regardless of format (e.g., information in text, reference list) | All observational work | Endorsement of promotion of intervention designed to prevent to treat condition (e.g., supplements, brain training games, foods) within the article | Article is dated within the last 5 years | No support of the patient-physician relationship | Balanced/cautious support (authors' claims are balanced by caution, includes statements of limitations and/or contrasting findings) | 16 |
| 35 | No | Organization takes authorship | Mention of expert source, research findings (though with insufficient information to identify the specific studies), links to various sites, | In vitro, animal models, or editorials | Unbiased information | Article is dated within the last 5 years | No support of the patient-physician relationship | Mainly supported (authors mainly support their claims but with more cautious vocabulary such as 'can reduce your risk' or 'may help prevent', no discussion of limitations | 15 |

| | | | | | | | | | |
|---|---|---|---|---|---|---|---|---|---|
| | | | advocacy body, or other | | | | | | |
| 36 | No | Organization takes authorship | No sources | None | Unbiased information | Article is dated within the last 5 years | No support of the patient-physician relationship | Mainly supported (authors mainly support their claims but with more cautious vocabulary such as 'can reduce your risk' or 'may help prevent', no discussion of limitations | 12 |
| 37 | No | All others indications of authorship | Reference to at least one identifiable scientific study, regardless of format (e.g., information in text, reference list) | All observational work | Unbiased information | Article is dated, but 5 years or older | No support of the patient-physician relationship | Balanced/cautious support (authors' claims are balanced by caution, includes statements of limitations and/or contrasting findings) | 21 |
| 38 | No | Author's name and qualification clearly stated | Reference to many identifiable scientific studies, regardless of format (in >50% of claims) | Meta-analyses, randomized controlled trials, clinical studies | Unbiased information | Article is dated, but 5 years or older | No support of the patient-physician relationship | Balanced/cautious support (authors' claims are balanced by caution, includes statements of limitations and/or contrasting findings) | 26 |
| 39 | No | All others indications of authorship | Mention of expert source, research findings (though with | In vitro, animal models, or editorials | Unbiased information | Article is dated within the last 5 years | No support of the patient-physician relationship | Balanced/cautious support (authors' claims are balanced by caution, includes statements of | 18 |

| | | | | | | | | | |
|---|---|---|---|---|---|---|---|---|---|
| | | | insufficient information to identify the specific studies), links to various sites, advocacy body, or other | | | | | limitations and/or contrasting findings) | |
| 40 | No | Organization takes authorship | Reference to at least one identifiable scientific study, regardless of format (e.g., information in text, reference list) | Meta-analyses, randomized controlled trials, clinical studies | Unbiased information | No date present | No support of the patient-physician relationship | Mainly supported (authors mainly support their claims but with more cautious vocabulary such as 'can reduce your risk' or 'may help prevent', no discussion of limitations | 18 |
| 41 | No | Organization takes authorship | Mention of expert source, research findings (though with insufficient information to identify the specific studies), links to various sites, advocacy body, or other | All observational work | Unbiased information | No date present | No support of the patient-physician relationship | Mainly supported (authors mainly support their claims but with more cautious vocabulary such as 'can reduce your risk' or 'may help prevent', no discussion of limitations | 14 |
| 42 | No | Organization takes authorship | Mention of expert source, research | None | Unbiased information | Article is dated, but 5 years or older | No support of the patient-physician relationship | Fully supported (authors fully and unequivocally support | 11 |

| | | | | | | | | | |
|---|---|---|---|---|---|---|---|---|---|
| | | | findings (though with insufficient information to identify the specific studies), links to various sites, advocacy body, or other | | | | | the claims, strong vocabulary such as 'cure', 'guarantee', and 'easy', mostly use of non-conditional verb tenses ('can, 'will'), no discussion of limitations) | |
| 43 | No | Organization takes authorship | Reference to many identifiable scientific studies, regardless of format (in >50% of claims) | Meta-analyses, randomized controlled trials, clinical studies | Unbiased information | Article is dated within the last 5 years | No support of the patient-physician relationship | Balanced/cautious support (authors' claims are balanced by caution, includes statements of limitations and/or contrasting findings) | 26 |
| 44 | No | Organization takes authorship | Reference to many identifiable scientific studies, regardless of format (in >50% of claims) | Meta-analyses, randomized controlled trials, clinical studies | Unbiased information | Article is dated within the last 5 years | No support of the patient-physician relationship | Balanced/cautious support (authors' claims are balanced by caution, includes statements of limitations and/or contrasting findings) | 26 |
| 45 | No | Organization takes authorship | Mention of expert source, research findings (though with insufficient information to | In vitro, animal models, or editorials | Unbiased information | Article is dated within the last 5 years | No support of the patient-physician relationship | Mainly supported (authors mainly support their claims but with more cautious vocabulary such as 'can reduce your risk' or 'may | 15 |

| | | | | | | | | | |
|---|---|---|---|---|---|---|---|---|---|
| | | | identify the specific studies), links to various sites, advocacy body, or other | | | | | help prevent', no discussion of limitations | |
| 46 | No | Organization takes authorship | Mention of expert source, research findings (though with insufficient information to identify the specific studies), links to various sites, advocacy body, or other | In vitro, animal models, or editorials | Unbiased information | Article is dated within the last 5 years | No support of the patient-physician relationship | Mainly supported (authors mainly support their claims but with more cautious vocabulary such as 'can reduce your risk' or 'may help prevent', no discussion of limitations | 15 |
| 47 | No | All others indications of authorship | Reference to at least one identifiable scientific study, regardless of format (e.g., information in text, reference list) | All observational work | Unbiased information | Article is dated within the last 5 years | No support of the patient-physician relationship | Balanced/cautious support (authors' claims are balanced by caution, includes statements of limitations and/or contrasting findings) | 22 |
| 48 | Yes | All others indications of authorship | Reference to at least one identifiable scientific study, | All observational work | Unbiased information | Article is dated within the last 5 years | No support of the patient-physician relationship | Mainly supported (authors mainly support their claims but with more cautious vocabulary | 19 |

| | | | | | | | | | |
|---|---|---|---|---|---|---|---|---|---|
| | | | regardless of format (e.g., information in text, reference list) | | | | | such as 'can reduce your risk' or 'may help prevent', no discussion of limitations | |
| 49 | No | All others indications of authorship | Reference to at least one identifiable scientific study, regardless of format (e.g., information in text, reference list) | All observational work | Unbiased information | Article is dated within the last 5 years | No support of the patient-physician relationship | Balanced/cautious support (authors' claims are balanced by caution, includes statements of limitations and/or contrasting findings) | 22 |
| 50 | No | Organization takes authorship | Mention of expert source, research findings (though with insufficient information to identify the specific studies), links to various sites, advocacy body, or other | All observational work | Unbiased information | Article is dated within the last 5 years | No support of the patient-physician relationship | Mainly supported (authors mainly support their claims but with more cautious vocabulary such as 'can reduce your risk' or 'may help prevent', no discussion of limitations | 16 |
| 51 | No | All others indications of authorship | Mention of expert source, research findings (though with insufficient information to | All observational work | Unbiased information | Article is dated within the last 5 years | No support of the patient-physician relationship | Balanced/cautious support (authors' claims are balanced by caution, includes statements of limitations and/or contrasting findings) | 19 |

| | | | | | | | | | |
|---|---|---|---|---|---|---|---|---|---|
| | | | identify the specific studies), links to various sites, advocacy body, or other | | | | | | |
| 52 | No | All others indications of authorship | Reference to at least one identifiable scientific study, regardless of format (e.g., information in text, reference list) | All observational work | Endorsement of promotion of intervention designed to prevent to treat condition (e.g., supplements, brain training games, foods) within the article | Article is dated within the last 5 years | No support of the patient-physician relationship | Balanced/cautious support (authors' claims are balanced by caution, includes statements of limitations and/or contrasting findings) | 16 |
| 53 | No | All others indications of authorship | Reference to at least one identifiable scientific study, regardless of format (e.g., information in text, reference list) | All observational work | Unbiased information | Article is dated within the last 5 years | No support of the patient-physician relationship | Balanced/cautious support (authors' claims are balanced by caution, includes statements of limitations and/or contrasting findings) | 22 |
| 54 | No | All others indications of authorship | No sources | None | Endorsement or promotion of educational products & services (e.g., books, care home services) | No date present | No support of the patient-physician relationship | Mainly supported (authors mainly support their claims but with more cautious vocabulary such as 'can reduce your risk' or 'may help prevent', no | 7 |

| | | | | | | | | | |
|---|---|---|---|---|---|---|---|---|---|
| | | | | | | | | discussion of limitations | |
| 55 | Yes | All others indications of authorship | Reference to at least one identifiable scientific study, regardless of format (e.g., information in text, reference list) | All observational work | Unbiased information | Article is dated within the last 5 years | No support of the patient-physician relationship | Balanced/cautious support (authors' claims are balanced by caution, includes statements of limitations and/or contrasting findings) | 22 |
| 56 | No | Organization takes authorship | Mention of expert source, research findings (though with insufficient information to identify the specific studies), links to various sites, advocacy body, or other | None | Unbiased information | No date present | No support of the patient-physician relationship | Mainly supported (authors mainly support their claims but with more cautious vocabulary such as 'can reduce your risk' or 'may help prevent', no discussion of limitations | 13 |
| 57 | No | All others indications of authorship | Mention of expert source, research findings (though with insufficient information to identify the specific studies), links | In vitro, animal models, or editorials | Unbiased information | Article is dated within the last 5 years | No support of the patient-physician relationship | Mainly supported (authors mainly support their claims but with more cautious vocabulary such as 'can reduce your risk' or 'may help prevent', no discussion of limitations | 15 |

| | | | | | | | | | |
|---|---|---|---|---|---|---|---|---|---|
| | | | to various sites, advocacy body, or other | | | | | | |
| 58 | No | All others indications of authorship | Mention of expert source, research findings (though with insufficient information to identify the specific studies), links to various sites, advocacy body, or other | All observational work | Unbiased information | Article is dated, but 5 years or older | No support of the patient-physician relationship | Balanced/cautious support (authors' claims are balanced by caution, includes statements of limitations and/or contrasting findings) | 18 |
| 59 | No | Organization takes authorship | No sources | None | Unbiased information | Article is dated within the last 5 years | No support of the patient-physician relationship | Fully supported (authors fully and unequivocally support the claims, strong vocabulary such as 'cure', 'guarantee', and 'easy', mostly use of non-conditional verb tenses ('can, 'will'), no discussion of limitations) | 9 |
| 60 | No | All others indications of authorship | Reference to at least one identifiable scientific study, regardless of format (e.g., | Meta-analyses, randomized controlled trials, clinical studies | Unbiased information | Article is dated within the last 5 years | No support of the patient-physician relationship | Balanced/cautious support (authors' claims are balanced by caution, includes statements of limitations and/or contrasting findings) | 23 |

| | | | | | | | | | |
|---|---|---|---|---|---|---|---|---|---|
| | | | information in text, reference list) | | | | | | |
| 61 | No | All others indications of authorship | Reference to many identifiable scientific studies, regardless of format (in >50% of claims) | Meta-analyses, randomized controlled trials, clinical studies | Unbiased information | Article is dated within the last 5 years | No support of the patient-physician relationship | Balanced/cautious support (authors' claims are balanced by caution, includes statements of limitations and/or contrasting findings) | 26 |
| 62 | No | Organization takes authorship | No sources | None | Unbiased information | Article is dated within the last 5 years | No support of the patient-physician relationship | Mainly supported (authors mainly support their claims but with more cautious vocabulary such as 'can reduce your risk' or 'may help prevent', no discussion of limitations | 12 |
| 63 | No | Organization takes authorship | No sources | None | Unbiased information | No date present | No support of the patient-physician relationship | Fully supported (authors fully and unequivocally support the claims, strong vocabulary such as 'cure', 'guarantee', and 'easy', mostly use of non-conditional verb tenses ('can, 'will'), no discussion of limitations) | 7 |
| 64 | No | Organization takes authorship | Reference to at least one identifiable | All observational work | Unbiased information | Article is dated within | No support of the patient-physician relationship | Balanced/cautious support (authors' claims are balanced | 22 |

| | | | | | | | | | |
|---|---|---|---|---|---|---|---|---|---|
| | | | scientific study, regardless of format (e.g., information in text, reference list) | | | the last 5 years | | by caution, includes statements of limitations and/or contrasting findings) | |
| 65 | No | Organization takes authorship | No sources | None | Unbiased information | No date present | No support of the patient-physician relationship | Fully supported (authors fully and unequivocally support the claims, strong vocabulary such as 'cure', 'guarantee', and 'easy', mostly use of non-conditional verb tenses ('can, 'will'), no discussion of limitations) | 7 |
| 66 | Yes | Organization takes authorship | Mention of expert source, research findings (though with insufficient information to identify the specific studies), links to various sites, advocacy body, or other | None | Unbiased information | Article is dated within the last 5 years | Support of the patient-physician relationship | Mainly supported (authors mainly support their claims but with more cautious vocabulary such as 'can reduce your risk' or 'may help prevent', no discussion of limitations | 16 |
| 67 | No | Organization takes authorship | Reference to at least one identifiable scientific | All observational work | Unbiased information | No date present | Support of the patient-physician relationship | Mainly supported (authors mainly support their claims but with more | 18 |

| | | | | | | | | |
|---|---|---|---|---|---|---|---|---|
| | | | study, regardless of format (e.g., information in text, reference list) | | | | | cautious vocabulary such as 'can reduce your risk' or 'may help prevent', no discussion of limitations | |
| 68 | No | Organization takes authorship | No sources | None | Unbiased information | No date present | No support of the patient-physician relationship | Fully supported (authors fully and unequivocally support the claims, strong vocabulary such as 'cure', 'guarantee', and 'easy', mostly use of non-conditional verb tenses ('can, 'will'), no discussion of limitations) | 7 |
| 69 | No | Organization takes authorship | Reference to many identifiable scientific studies, regardless of format (in >50% of claims) | Meta-analyses, randomized controlled trials, clinical studies | Unbiased information | Article is dated within the last 5 years | No support of the patient-physician relationship | Balanced/cautious support (authors' claims are balanced by caution, includes statements of limitations and/or contrasting findings) | 26 |
| 70 | No | All others indications of authorship | Mention of expert source, research findings (though with insufficient information to identify the specific studies), links | None | Unbiased information | Article is dated within the last 5 years | No support of the patient-physician relationship | Balanced/cautious support (authors' claims are balanced by caution, includes statements of limitations and/or contrasting findings) | 18 |

| | | | | | | | | | |
|---|---|---|---|---|---|---|---|---|---|
| | | | to various sites, advocacy body, or other | | | | | | |
| 71 | No | All others indications of authorship | Reference to at least one identifiable scientific study, regardless of format (e.g., information in text, reference list) | All observational work | Unbiased information | Article is dated within the last 5 years | No support of the patient-physician relationship | Balanced/cautious support (authors' claims are balanced by caution, includes statements of limitations and/or contrasting findings) | 22 |
| 72 | No | Organization takes authorship | Mention of expert source, research findings (though with insufficient information to identify the specific studies), links to various sites, advocacy body, or other | None | Endorsement or promotion of educational products & services (e.g., books, care home services) | No date present | Support of the patient-physician relationship | Mainly supported (authors mainly support their claims but with more cautious vocabulary such as 'can reduce your risk' or 'may help prevent', no discussion of limitations | 11 |
| 73 | No | All others indications of authorship | Mention of expert source, research findings (though with insufficient information to identify the | None | Unbiased information | Article is dated within the last 5 years | No support of the patient-physician relationship | Balanced/cautious support (authors' claims are balanced by caution, includes statements of limitations and/or contrasting findings) | 18 |

| | | | | | | | | | |
|---|---|---|---|---|---|---|---|---|---|
| | | | specific studies), links to various sites, advocacy body, or other | | | | | | |
| 74 | No | Author's name and qualification clearly stated | No sources | None | Unbiased information | Article is dated within the last 5 years | No support of the patient-physician relationship | Mainly supported (authors mainly support their claims but with more cautious vocabulary such as 'can reduce your risk' or 'may help prevent', no discussion of limitations | 13 |
| 75 | No | Author's name and qualification clearly stated | Mention of expert source, research findings (though with insufficient information to identify the specific studies), links to various sites, advocacy body, or other | In vitro, animal models, or editorials | Unbiased information | Article is dated within the last 5 years | No support of the patient-physician relationship | Mainly supported (authors mainly support their claims but with more cautious vocabulary such as 'can reduce your risk' or 'may help prevent', no discussion of limitations | 16 |
| 76 | No | Author's name and qualification clearly stated | Reference to at least one identifiable scientific study, regardless of | Meta-analyses, randomized controlled trials, clinical studies | Unbiased information | Article is dated within the last 5 years | No support of the patient-physician relationship | Balanced/cautious support (authors' claims are balanced by caution, includes statements of | 24 |

| | | | | | | | | | |
|---|---|---|---|---|---|---|---|---|---|
| | | | format (e.g., information in text, reference list) | | | | | limitations and/or contrasting findings) | |
| 77 | No | All others indications of authorship | Reference to at least one identifiable scientific study, regardless of format (e.g., information in text, reference list) | All observational work | Unbiased information | Article is dated, but 5 years or older | No support of the patient-physician relationship | Balanced/cautious support (authors' claims are balanced by caution, includes statements of limitations and/or contrasting findings) | 21 |
| 78 | No | Organization takes authorship | Mention of expert source, research findings (though with insufficient information to identify the specific studies), links to various sites, advocacy body, or other | None | Unbiased information | Article is dated within the last 5 years | No support of the patient-physician relationship | Fully supported (authors fully and unequivocally support the claims, strong vocabulary such as 'cure', 'guarantee', and 'easy', mostly use of non-conditional verb tenses ('can, 'will'), no discussion of limitations) | 12 |
| 79 | No | All others indications of authorship | Reference to at least one identifiable scientific study, regardless of format (e.g., information in | Meta-analyses, randomized controlled trials, clinical studies | Unbiased information | Article is dated within the last 5 years | No support of the patient-physician relationship | Balanced/cautious support (authors' claims are balanced by caution, includes statements of limitations and/or contrasting findings) | 23 |

| | | | | | | | | | |
|---|---|---|---|---|---|---|---|---|---|
| | | | text, reference list) | | | | | | |
| 80 | No | All others indications of authorship | Reference to at least one identifiable scientific study, regardless of format (e.g., information in text, reference list) | Meta-analyses, randomized controlled trials, clinical studies | Unbiased information | Article is dated within the last 5 years | No support of the patient-physician relationship | Balanced/cautious support (authors' claims are balanced by caution, includes statements of limitations and/or contrasting findings) | 23 |
| 81 | No | All others indications of authorship | Reference to at least one identifiable scientific study, regardless of format (e.g., information in text, reference list) | All observational work | Unbiased information | Article is dated, but 5 years or older | No support of the patient-physician relationship | Balanced/cautious support (authors' claims are balanced by caution, includes statements of limitations and/or contrasting findings) | 21 |
| 82 | No | All others indications of authorship | Reference to at least one identifiable scientific study, regardless of format (e.g., information in text, reference list) | All observational work | Unbiased information | Article is dated within the last 5 years | No support of the patient-physician relationship | Balanced/cautious support (authors' claims are balanced by caution, includes statements of limitations and/or contrasting findings) | 22 |
