# Supplementary material for: The Quality, Readability, and Accuracy of the Information on Google About Cannabis and Driving: Quantitative Content Analysis
Source: JMIR Infodemiology. 2023 May 2;3:e43001. doi: 10.2196/43001 (PMC10189625; doi:10.2196/43001)
Supplement: Multimedia Appendix 5 [file infodemiology_v3i1e43001_app5.pdf]

Multimedia Appendix 5

Table S4 : The readability scores of included web pages

ID	Readability scores			
	**Flesch-Kincaid Grade Level**	**Gunning Fog Index**	**SMOG Index**	**Flesch Reading Ease**
1	11.2	14.7	13.9	42.8
2	10.8	12.8	13.8	43.9
3	11.8	13.6	13.6	35.3
4	8.5	11.6	12	53.3
5	7.8	9.9	10.8	58.4
6	9.1	11	12.3	52.3
7	12.1	13.2	13.7	37.4
8	5.1	4.6	8	67.7
9	10.8	13.5	13.7	42.9
10	7.9	5.1	7.9	43.9
11	9	11.8	12.2	53.2
12	8.1	10.5	10.5	52.2
13	11	10.9	12.7	38.2
14	10	12.9	13.2	51.7
15	11.1	13	13.3	47.9
16	9	11.8	11.2	47
17	8.4	8.8	10.6	51.7
18	9.1	9.1	11.3	11.3
19	10.5	12.2	12.5	50.2
20	12.2	13.1	13.5	29
21	10.5	11	12.3	38
22	16.5	19.7	17	10.7
23	11.8	14.3	14	46
24	7.6	8.5	10	56.1
25	12.7	11.8	14.4	32.8
26	11.2	13.2	13.1	45.2
27	10	11.5	12.5	43
28	13.1	14.7	14.5	36
29	16.2	17.9	17.4	22.8
30	6.7	8.5	9.8	60.1
31	8.2	10.1	11.4	52.8
32	10.8	12.4	12.8	52.4
33	11.4	12.7	13.8	43.6
34	10.5	12.5	12.8	47.7
35	8.1	10.7	11.3	57.9
36	7.7	10.2	10.9	64.7
37	9.7	11.3	12.2	57.2
38	17.3	20.5	18.4	13.3
39	10.3	12.8	12.9	55.5
40	8.4	10.7	11.5	54.8
41	9.8	12	12.4	49.6
42	6.7	9.1	9.9	71.1

43	8.6	9.8	10.9	47.3
44	10.1	13.1	13	47.1
45	8.5	10.1	11.8	57
46	8.4	9.9	11	58.5
47	8.7	10.6	11.7	53.4
48	7	7.8	10.1	60.7
49	9.4	11.1	12.1	52.9
50	6.8	4.9	9.5	60.2
51	8.4	10.1	11.3	54.7
52	9.4	10.8	11.9	51.6
53	9.3	10.3	11.9	51.9
54	10.3	12.1	13.1	49.8
55	13.4	15.6	15	37.7
56	6.2	7.6	9.5	69
57	7	9.8	10.6	69.8
58	11.4	13.2	13.8	43.4
59	4.3	7	8.4	78.3
60	9.9	11.6	12.3	51.3
61	10.1	12.4	12.4	53.5
62	8.1	10.5	11.6	60.8
63	6.8	7.9	9.9	62.8
64	12.5	15	15	35.7
65	7.2	10.4	11.3	61.8
66	7.9	9.2	10.7	59.5
67	10.3	12.7	13.2	47.2
68	9.2	11.5	12.2	53.1
69	12	13.1	13.7	38.3
70	10	12.6	12.9	54
71	9.8	11.7	12.4	52.2
72	10.4	12.3	12.4	53.6
73	10.4	12.6	12.7	55
74	10.1	11.5	12.2	49.9
75	10.4	13	12.6	55.4
76	9.7	10.3	12.3	52.8
77	8.5	8.7	11.4	57.4
78	4.8	4.6	7.4	66.3
79	10.9	12.5	13.3	51.3
80	10.6	12.8	12.7	55.3
81	9.2	10.6	11.5	56.2
82	13.4	15.5	14.7	41.8
