# Supplementary material for: The Quality, Readability, and Accuracy of the Information on Google About Cannabis and Driving: Quantitative Content Analysis
Source: JMIR Infodemiology. 2023 May 2;3:e43001. doi: 10.2196/43001 (PMC10189625; doi:10.2196/43001)
Supplement: Multimedia Appendix 6 [file infodemiology_v3i1e43001_app6.pdf]

Multimedia Appendix 6

Table S5 : Examples of quotations used for accuracy assessment

Cannabis topics/ Accuracy scale	Accurate	Mixed accuracy	Inaccurate
Cannabis consumption	• "Cannabis can impair each person differently. The impairment on individuals can depend on: The method of consumption if it was smoked, inhaled, or ingested; The quantity of cannabis consumed; The recency of use; The frequency of use; and The variety of cannabis and its THC levels, including cannabis prescribed for medical use"(ID#6)<br><br>• "When cannabis is smoked, tetrahydrocannabinol (THC) is rapidly transferred into the blood from the lungs, reaching a peak within minutes of smoking and dissipating slowly over several hours"(ID#44)		• "Studies have shown that actual impairment after consuming cannabis subsides after two and a half hours AND Remember that medication, alcohol, gender, age, diet and lifestyle can all affect the rate at which you eliminate THC"(ID#45)
Prevalence of DUIC	• "Driving after using cannabis is relatively common in Canada" (ID#5)<br><br>• "Cannabis is the most common drug found in drivers aged 16 to 19" (ID#17)		• "40% of Canadian drivers killed in vehicle crashes tested positive for drugs. This figure exceeds the percentage of drivers that tested positive for alcohol (33%). Unfortunately, these statistics indicate that the legalization of cannabis has caused more cannabis-related road accidents" (ID#8)
Effects of cannabis impairment on driving ability	• "Some of these effects include: reduced ability to divide attention, poor time and space management, reduced ability to allocate concentration AND Just a small amount of alcohol mixed with cannabis	• "Large doses of marijuana can affect perception and psychomotor performance, changes that could impair driving ability. But in driving studies, marijuana produces little or no car-handling impairment AND Mixing	

	considerably increases the negative effects on driving skills"(ID#17)<br><br>• "Marijuana causes problems with memory, attention and problem-solving in kids. It also affects their judgment, concentration, reaction time and coordination AND Mixing marijuana and alcohol adds to the crash risk, especially for teens"(ID27)	marijuana and alcohol together amplifies potential risks"(ID#24)<br><br>• "Psychomotor coordination is not impaired by dope, but judgment apparently is"(ID#46)	
Risk of collision	• "[Cannabis can] increase your chance of a crash"(ID#18)<br><br>• "Research from the National Institute of Drug Abuse suggests that even after the high ends, as long as there is THC in someone's system, the person is twice as likely to get into an accident"(ID#77)	• "generally accepted results report a two-fold risk of a motor vehicle crash among drivers with any THC in the bloodstream"(ID#31)<br><br>• "cannabis use by drivers exceeds that of alcohol among nighttime drivers and cannabis is among the most frequently found drugs in drivers involved in serious crashes"(ID#55)	• "For some people, marijuana may play a role in bad driving, but the overall rate of highway accidents does not appear to be significantly affected by marijuana's widespread use"(ID#24)<br><br>• "Yet there is no cold, hard fact that says marijuana causes accidents. In fact, all the data we can lay our hands on tell us marijuana not only doesn't impair driving ability, it can sometimes improve"(ID#46)
Detection by law	• "Police have tools and tests to detect impaired drivers, including roadside drug screening equipment and sobriety tests"(ID#4)<br><br>• "Police are trained to detect if you are driving under the influence of a drug and enforce drug-impaired driving laws using: SFST and DRE. AND Trained police officers and Drug Recognition Experts can determine if you are under the influence of a drug and can charge you with impaired driving"(ID#6)	• "enforcers and scientists agree that implementing laws on marijuana use is complicated because of the variable effects of the drug and difficulties defining a legal limit"(ID#62)<br><br>• "This is what it's called when a driver is under the influence of any substance, is stopped or pulled over, given a field sobriety test and fails, meaning that the arresting officer judges them to be unable to responsibly operate their vehicle.<br><br>• With alcohol, the blood-alcohol level in a person's	• "Thus, even though it is possible that cannabis impairs driving ability to some extent, there are currently no reliable means to test or measure whether a driver was actually impaired"(ID#20)<br><br>• "The NIJ report states, "the results of the study seem to indicate that both tests could be completely unreliable factors in determining whether someone is capable of driving," when it comes to cannabis"(ID#51)

		system is directly indicative of their level of impairment. With a person driving high on weed, there's no such test that indicates that they're impaired because of marijuana use"(ID#79)	
